# Utility of EST-derived SSR in cultivated peanut (*Arachis hypogaea *L.) and *Arachis *wild species

**DOI:** 10.1186/1471-2229-9-35

**Published:** 2009-03-24

**Authors:** Xuanqiang Liang, Xiaoping Chen, Yanbin Hong, Haiyan Liu, Guiyuan Zhou, Shaoxiong Li, Baozhu Guo

**Affiliations:** 1 Crops Research Institute, Guangdong Academy of Agricultural Sciences, Wushan 510640, Guangzhou, PR China; 2 USDA-ARS, Crop Protection and Management Research Unit, Tifton, Georgia, USA

## Abstract

**Background:**

Lack of sufficient molecular markers hinders current genetic research in peanuts (*Arachis hypogaea *L.). It is necessary to develop more molecular markers for potential use in peanut genetic research. With the development of peanut EST projects, a vast amount of available EST sequence data has been generated. These data offered an opportunity to identify SSR in ESTs by data mining.

**Results:**

In this study, we investigated 24,238 ESTs for the identification and development of SSR markers. In total, 881 SSRs were identified from 780 SSR-containing unique ESTs. On an average, one SSR was found per 7.3 kb of EST sequence with tri-nucleotide motifs (63.9%) being the most abundant followed by di- (32.7%), tetra- (1.7%), hexa- (1.0%) and penta-nucleotide (0.7%) repeat types. The top six motifs included AG/TC (27.7%), AAG/TTC (17.4%), AAT/TTA (11.9%), ACC/TGG (7.72%), ACT/TGA (7.26%) and AT/TA (6.3%). Based on the 780 SSR-containing ESTs, a total of 290 primer pairs were successfully designed and used for validation of the amplification and assessment of the polymorphism among 22 genotypes of cultivated peanuts and 16 accessions of wild species. The results showed that 251 primer pairs yielded amplification products, of which 26 and 221 primer pairs exhibited polymorphism among the cultivated and wild species examined, respectively. Two to four alleles were found in cultivated peanuts, while 3–8 alleles presented in wild species. The apparent broad polymorphism was further confirmed by cloning and sequencing of amplified alleles. Sequence analysis of selected amplified alleles revealed that allelic diversity could be attributed mainly to differences in repeat type and length in the microsatellite regions. In addition, a few single base mutations were observed in the microsatellite flanking regions.

**Conclusion:**

This study gives an insight into the frequency, type and distribution of peanut EST-SSRs and demonstrates successful development of EST-SSR markers in cultivated peanut. These EST-SSR markers could enrich the current resource of molecular markers for the peanut community and would be useful for qualitative and quantitative trait mapping, marker-assisted selection, and genetic diversity studies in cultivated peanut as well as related *Arachis *species. All of the 251 working primer pairs with names, motifs, repeat types, primer sequences, and alleles tested in cultivated and wild species are listed in Additional File 1.

## Background

Cultivated peanut (*Arachis hypogaea *L.) is grown on 25.5 million hectares with a total global production of about 35 million tons. It is an allotetraploid (2n = 4× = 40) and belongs to *Arachis *genus, which can be grouped into nine sections and includes approximately 80 species [1]. A large amount of morphological and agronomic variation is evident among accessions of cultivated peanuts, but extremely low levels of polymorphism were observed using restriction fragment length polymorphism (RFLP), randomly amplified polymorphic DNA (RAPD) and amplified fragment length polymorphisms (AFLP) [2-5]. Only simple-sequence repeats (SSRs) showed a potential for use in genetic studies of cultivated peanuts [6-11]. However it is expensive, labor-intensive and time-consuming to develop SSR markers from genomic DNA libraries [12]. To date, the number of available SSRs is grossly inadequate for mapping studies. Although several peanut genetic maps have been published [13-16], the existing maps do not have sufficient markers to be highly useful for genetic studies. Thus, there is great need for development of novel SSR markers.

Recently, EST-SSRs have received much attention as the increasing amounts of ESTs being deposited in databases for various plants [17-19]. EST-SSR can be rapidly developed from EST database by data mining at low cost, and due to their existence in transcribed region of genome, they can lead to the development of gene-based maps which may help to identify candidate function genes and increase the efficiency of marker-assisted selection [20]. In addition, EST-SSRs show a higher level of transferability to closely related species than genomic SSR markers [17,21] and can be served as anchor markers for comparative mapping and evolutionary studies [22]. Similar advantages of EST-SSRs have been reported for a number of plant species, such as grape [17], Medicago species [23], soybean [24], sugarcane [25], maize [18,19,24,26], rice [18,27-29], rye [29-31], and wheat [27,32,33], indicating that EST-SSR markers have potential for use in peanut genetic studies.

In peanut, only two studies described the development of EST-SSR in cultivated peanut and wild species [34,35]. Luo et al (2005) developed 44 EST-SSR markers from 1,350 cultivated peanut ESTs, nine of which exhibited polymorphism among 24 cultivated peanut lines. Proite et al (2007) developed 188 EST-SSRs from 8,785 *A. stenosperma *(*Arachis *species) ESTs, of which, 21 were polymorphic for an AA genome mapping population and 4 for a range of cultivated peanut genotypes. In this study, we screened a much larger number of ESTs (24, 238) from cultivated peanut with the following objectives: (1) to analyze the frequency and distribution of SSRs in transcribed regions of cultivated peanut genome; (2) to assess the validity of developed EST-SSR markers for detection of the polymorphism in cultivated peanut genotypes and their transferability to related wild species; (3) to develop new EST-SSR markers for both cultivated peanut and wild species.

## Results

### Type and frequency of peanut EST-SSRs

A total of 24,238 ESTs with an average length of 550 bp were used to evaluate the presence of SSR motifs. To eliminate redundant sequences and improve the sequence quality, the TIGR Gene Indices Clustering Tools (TGICL) [36] was employed to obtain consensus sequences from overlapping clusters of ESTs. A cluster was defined here as a group of overlapping EST sequences (at least 50 nucleotides with 90% identity and unmatched length less than 20 nucleotides). Totally, 11,431 potential unique ESTs including 1,434 contigs and 9,997 singletons were generated. As shown in Table 1, a total of 881 SSRs were identified from 780 unique ESTs, with an average of one SSR per 7.3 kb. Of those, 85 (about 10.9%) ESTs contained more than one SSR and 59 (about 7.6%) were compound SSRs that have more than one repeat type. Analysis of SSR motifs revealed that the proportion of SSR unit sizes was not evenly distributed. The occurrences of different repeat units were tri- (63.9%), di- (32.7%), tetra- (1.7%), penta- (0.7%), and hexa-nucleotide (1.0%). The mean SSR length of each unit varied between 18 and 37 bp. The overall average SSR length was 20 bp with a maximum of 86 bp di-nucleotide repeat (AG/CT). A total of 27 SSR motifs were listed in Table 2. The AG/CT was the most frequent motif and accounted for 27.7%, followed by AAG/TTC (17.37%), AAT/TTA (11.9%), ACC/TGG (7.7%), ACT/TGA (7.26%) and AT/TA (6.3%). The remaining motifs presented a frequency of 23.3%. GC-only repeat was not observed.

**Table 1:** Summary of SSR search after sequences assembled and categorized

	Contigs(bp)	Singlets(bp)	Total (bp)
EST after assembled	1434(12372129)	9997(5197116)	11431(6434245)
Identifed SSRs	180	701	881
ESTs having SSRs	156	624	780
ESTs having more than 1SSR	19	66	85
Compound SSRs	16	43	59
Bi-type	73	215	288
Tri-type	98	465	563
Tetra-type	7	8	15
Penta-type	1	5	6
Hexa-type	1	8	9

**Table 2:** Occurrence and number of repeats of 27 SSR motifs in cultivated peanut (*Arachis hypogaea *L.)

**Repeats**	**Number of repeat units**										**Total repeat**
		
	**5**	**6**	**7**	**8**	**9**	**10**	**11**	**12**	**13**	**Above**	
AC/GT	-	-	6	2	3	2		1			14
AG/CT	-	-	56	43	31	11	13	15	2	47	218
AT/AT	-	-	15	11	5	3	2	2	1	17	56
AAC/GTT	22	7	1	2	5						37
AAG/CTT	71	44	13	10	7	4	2			2	153
AAT/ATT	54	26	8	3	3	2		1	3	5	105
ACC/GGT	31	22	9	5	1						68
ACG/CTG	10	4	2	1							17
ACT/ATG	35	17	9	1			2				64
AGC/CGT	17	8	1	2	1						29
AGG/CCT	19	8	1	1							29
AGT/ATC	25	11	4	2		1					43
CCG/CGG	13	3	1	1							18
AAAG/CTTT	3	3		1							7
AAAT/ATTT	2										2
AATC/AGTT	2	1									3
AATT/AATT	1										1
ACAT/ATGT	1	1									2
AAAAG/CTTTT	1										1
AAAAT/ATTTT	2										2
AGTAT/ATATC	3										3
AAAAAG/CTTTTT	1										1
AAGACG/CTGCTT	2										2
AATAGT/ATCATT				1							1
AATGAT/ACTATT			1	2							3
AGCAGT/ATCGTC	1										1
AGCTCC/AGGTCG	1										1

Total	317	155	127	88	56	23	19	19	6	71	881

### Primer design and validation

Among the 780 SSR-containing ESTs, 490 did not qualify for primer design as the flanking sequences were too short or poor quality. Therefore, only 290 primer pairs were designed and employed for validation of genic SSR markers (Table 3). Of these EST-SSRs, 65, 178 and 47 were observed in 5' untranslated terminal regions (UTR), translated regions and 3' UTR, respectively. After optimization, 251 primer pairs (86.5%) were successfully amplified in all cultivated peanut and wild species tested (Table 3), while the rest failed to give PCR products at various annealing temperature and Mg^2+ ^concentrations. Out of 251 working primer pairs, 182 amplified the expected size of amplicons, 41 yielded PCR products larger than expected, revealing that an intron is inside the amplicons, and the amplified products of the remaining 28 primer pairs were smaller than expected, suggesting the occurrence of deletion within the genomic sequences or a lack of specificity (Additional File 1).

**Table 3:** Characteristics of cultivated peanut (*Arachis hypogaea *L.) EST-SSR and efficiency of markers development

**Motif**	**No. of EST-SSRs**	**No. of designed primers**	**No. amplified EST-SSRs (%)**		**No. polymorphic EST-SSRs (%)**	
			
			**Cultivated peanut**	**Wild species**	**Cultivated peanut**	**Wild species**
Di	288	55	42	42	10	34
AC/GT	14	6	5	5	2	4
AG/CT	218	39	29	29	8	24
AT/AT	56	10	8	8	0	6
Tri	563	221	196	196	14	174
AAC/GTT	37	14	11	11	0	9
AAG/CTT	153	59	51	51	2	43
AAT/ATT	105	27	24	24	4	23
ACC/GGT	68	32	29	29	2	28
ACG/CTG	17	4	3	3	0	3
ACT/ATG	64	26	24	24	2	21
AGC/CGT	29	10	9	9	3	9
AGG/CCT	29	16	15	15	0	11
AGT/ATC	43	23	21	21	1	19
CCG/CGG	18	10	9	9	0	8
Tetra	15	5	5	5	1	5
AAAG/CTTT	7	1	1	1	0	1
AAAT/ATTT	2	2	2	2	1	2
AATC/AGTT	3	1	1	1	0	1
AATT/AATT	1	0	0	0	0	0
ACAT/ATGT	2	1	1	1	0	1
Penta-type	6	3	3	3	0	3
AAAAG/CTTTT	1	1	1	1	0	1
AAAAT/ATTTT	2	0	0	0	0	0
AGTAT/ATATC	3	2	2	2	0	2
Hexa-type	9	6	5	5	1	5
AAAAAG/CTTTTT	1	1	1	1	0	1
AAGACG/CTGCTT	2	1	1	1	1	1
AATAGT/ATCATT	2	2	1	1	0	1
AATGAT/ACTATT	3	1	1	1	0	1
AGCAGT/ATCGTC	1	0	0	0	0	0
AGCTCC/AGGTCG	1	1	1	1	0	1

Total	881	290	251	251	26	221

### EST-SSR polymorphism

In the present study, 251 valid EST-SSR primer pairs were used for assessment of the polymorphism among cultivated and wild *Arachis *species. Within cultivated peanuts, 26 (10.3%) EST-SSRs exhibited polymorphism (Table 3). A total of 55 alleles were detected and the average number of alleles per SSR marker was 2.1 with a range of 2–4 alleles based on the dominant scoring of the SSR bands characterized by the presence or absence of a particular band (Additional File 1). The PIC values ranged from 0.09 to 0.69 with an average value of 0.33. The greatest variation of SSR alleles was found for EM-78, which interacted with 4 alleles in 22 cultivated peanuts genotypes and the PIC value was 0.69.

The polymorphism of 251 cultivated peanut-derived EST-SSR in 16 accessions of wild species was evaluated. The results showed that 221 of 251 EST-SSR loci (88%) were polymorphic (Table 3), with a total of 867 alleles (Additional File 1). Allelic diversity was estimated for those polymorphic EST-SSR markers. The number of alleles detected among 16 wild species ranged from 2 to 9, with an average of 3.9 alleles per locus (Additional File 1). A maximum of 9 alleles were observed for primer EM-71. The PIC values varied between 0.594 and 0.820 with an average value of 0.721.

### Sequence comparison of SSR bands

**Figure 1:**
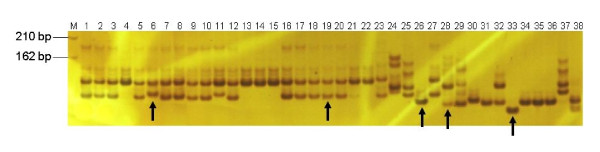
**Polyacrylamide gel electrophoresis patterns of microsatellite alleles amplified with the primer EM-31**. The bands indicated by the arrows were sequenced. M represents the DNA molecular weight marker, and 1–38 represent PI 393531 (1), PI 390693 (2), Qiongshanhuasheng (3), Liaoningsilihong (4), Dedou (5), Guangliu (6), Sanyuening (7), Yueyou 20 (8), Spancross (9), Tennessee Red (10), Xiaoliuqiu (11), Yangjiangpudizan (12), Xihuagoudo (13), Padou (14), Bo-50 (15), Yingdejidouzai (16), Heyuanbanman (17), Tuosunxiaohuasheng (18), Sunoleic 97R (19), Tifrunner (20), Georgia Green (21), NC940-22 (22), *A. villosa *(23), *A. stenosperma *(24), *A. correntina *(25), *A. cardenasii *(26), *A. magna *(27), *A. duranensis *(28), *A. chacoensis *(29), *A. batizocoi *(30), *A. helodes *(31), *A. monticola *(32), *A. pintoi *(33), *A. paraguariensis *(34), *A. pusilla *(35), *A. rigonii *(36), *A. appressipila *(37), *A. glabrata *(38).

**Figure 2:**
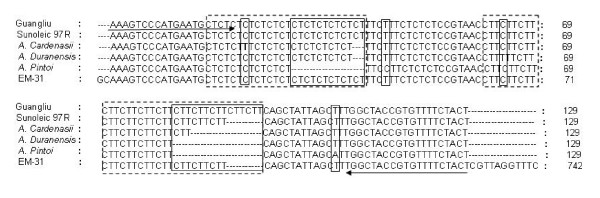
**Alignment of sequences obtained from five SSR bands amplified by EM-31 primers and original SSR-derived EST sequence(EM-31)**. Primer sequences are indicated by underlined arrows. Repetitive sequences are indicated in dashed box. Point mutations and indel regions are marked by box with solid line.

For further understanding of the EST-SSR polymorphism at the nucleotide level, the amplified products of primer EM-31 from two genotypes of cultivated peanuts and three accessions of wild species were cloned and sequenced (Figure 1, Figure 2). All the sequenced alleles from both cultivars and wild species were highly identical to the original locus (EST sequence) from which the EST-SSR marker EM-31 was mined. Sequence alignment showed that all the primer-binding regions are highly conserved. Allelic diversity could be attributed mainly to differences in repeat type and length in the microsatellite regions, although some variations such as repeat number or insertions of additional motifs were observed in the microsatellite regions. In addition, a few single base substitutions were observed in the microsatellite flanking regions. Out of them, one occurred in *A. cardenasii*, one in *A. duranensis*, and two in *A. pintoi*.

## Discussion

### Frequency and distribution of EST-SSRs

The frequency of SSRs in SSR-ESTs more accurately reflects the density of SSRs in the transcribed region of the genome. However, random sequencing within cDNA libraries usually resulted in a high proportion of redundant ESTs. In this study, to reduce the dataset size and avoid overestimation of the EST-SSR frequency, SSR search were performed following redundancy elimination. A total of 11,432 potential unique EST sequences (about 6.4 Mb) were used for SSR search and 6.8% (780) of ESTs contained specified SSR motifs, generating 881 unique SSRs. This is a relatively higher abundance of SSRs for peanut ESTs, compared to the previous reports for maize (1.4%), barley (3.4%), wheat (3.2%), soyghum (3.6%), rice (4.7%) [18], *Medicago truncatula *(3.0%) [23] and wild *Arachis *species [34]. The different abundance of SSRs was known to be dependent on the SSR search criteria, the size of the dataset, the database-mining tools and different species [22]. In this work, the frequency of occurrence for EST-derived SSRs was one EST-SSR in every 7.3 kb. In previous reports, an EST-SSR occurs every 13.8 kb in *Arabidopsis thaliana*, 3.4 kb in rice, 8.1 kb in maize, 7.4 kb in soybean, 11.1 kb in tomato, 20.0 kb in cotton and 14.0 kb in poplar [37]. The variations of frequencies among different studies were mainly due to the criteria used to identify SSR in the database mining.

In earlier reports, tri-nucleotide repeats were generally the most common motif found in both monocots [22] and dicots [23]. During the process of mining EST-SSRs in the various plant species, tri-nucleotide was also observed to be most frequent [26], regardless of the EST-SSR search criteria. Until now, only one report described that di-nucleotide repeats were most abundant followed by tri- or mono-nucleotide repeats in dicots [38]. In the present investigation, tri-nucleotide repeat was found to be abundant followed by di-nucleotide. In term of single SSR motif, the di-nucleotide motif (AG/TC)_*n *_was highest frequent [18,39]. Among the di-nucleotide motifs, the two most dominant motif types were AG and AT, representing an average frequency of 24.7% and 6.4%, respectively. This was in agreement with recent studies in cultivated peanut (*Arachis hypogaea *L.) [35] and wild *Arachis *species [34]. In this work, the AAG with 17.4% of frequency following di-nucleotide motif AG was the most abundant in the ten tri-nucleotide motifs. In other plant species, the most frequent tri-nucleotide repeat motifs were (AAC/TTG)_*n *_in wheat, (AGG/TCC)_*n *_in rice, (CCG/GGC)_*n *_in maize, (AAG/TTC)_*n *_in soybean, and (CCG/GGC)_*n *_in barley and sorghum [18,19,39,40]. The previous studies of *Arabidopsis *[37] and soybean [24] also suggested that the tri-nucleotide AAG motif may be common motif in dicots. In contrast, the abundance of the tri-nucleotide CCG repeat motif was favored overwhelmingly in cereal species [18,19,32] and also considered as a specific feature of monocot genome, which may be due to increasing the G + C content [26].

### Validation and polymorphism of EST-SSR markers

In this study, a total of 290 designed primer pairs were used for validation of the EST-SSR markers. Of these, 251 (86.5%) yielded amplicons in both cultivated peanut and wild species. This result was similar to previous studies in which a success rate of 60–90% amplification has been reported [21,25,40-42]. In those studies, they also reported a similar success rate of amplification for both genomic SSRs and EST-SSRs. However, EST-SSRs were reported to be less polymorphic than genomic SSRs in crop plants due to greater DNA sequence conservation in transcribed regions [17,28,43-46]. Previous studies highlighted the fact that EST-SSR markers have higher transferability and better applicability than genomic SSR markers [17,47-49]. In addition to high transferability, EST-SSRs were good candidates for the development of conserved orthologous markers for genetic analysis and breeding of different species [22]. Pervious reports showed that the transferability of EST-SSRs from one species to another ranged from 40–89% [21,23,24,27,29,40,41,50,51]. Our results indicated that 100% of EST-SSR amplifiable primers for cultivated peanut can produce amplicons in *Arachis *wild species.

In the present investigation, the mean percentage of polymorphic loci of EST-SSR markers was 9.96% in cultivated peanuts. This value was lower than those of genomic SSR found in earlier studies [7,12,47], but higher than the percentage of polymorphic loci in cultivated peanut observed using RAPD (6.6%) [5] and AFLP (6.7%) [4]. No major difference was observed in terms of allele numbers and PIC values for the EST-SSR markers among the cultivated genotypes, while significant difference was observed among wild species. Therefore, the low level of EST-SSR polymorphism detected in cultivated peanuts may be compensated by their higher potential for cross-species transferability to wild species. In the present study, 100% transferability of EST-SSR with 86.6% polymorphism from cultivated peanut to wild *Arachis *species was observed. The value is higher than that of genomic SSR cross-transferability [10]. The high level of transferability indicated that these markers would be highly effective for molecular study of *Arachis *species. Since current molecular markers display a low level of genetic polymorphism in cultivated peanuts [2-4,6,52,53], it is difficult to construct a high-density genetic linkage map for cultivated peanut which could be used in breeding programs. However, a genetic map constructed using wild species together with transferable molecular markers derived from cultivated peanuts would contribute to understanding the introgression of genes from wild species to cultivated peanuts [10,13]. Therefore, the development of a set of transferable EST-SSR markers from cultivated peanuts will be a great benefit to construct a high-density genetic map of wild species. The map would allow the identification of markers, especially transferable EST-SSR markers, associated with resistance or other agronomic traits in wild species, and in turn, help to discover corresponding markers or genes in cultivated peanuts.

Additionally, a comparison of sequences of cross-species amplicons generated by primer EM-31 further confirmed the conservation and transferability of the developed EST-SSR loci. In general, the amplified regions were found to be similar to the original peanut EST sequences from which the SSRs were developed and their comparisons across species (Figure 2) correlated the observed 'cross-species alleles' precisely with the expected length variations. Furthermore, in addition to the variation of the number of SSR repeat, the allele sequences also indicated that a few additional point mutation in the SSR motifs flanking regions. Similar variation has been reported in earlier studies [39,47,54,55]. This phenomenon is supposed to be the innate evolving nature of the genome, and thus can be indicative of the evolutionary relationships of the tested taxa [47].

## Conclusion

EST-SSR markers developed in this study will complement the genomic SSR markers and provide a valuable resource for linkage mapping, gene and QTL identification, and marker-assisted selection in peanut genetic study. Since these markers were developed based on expressed sequence and they are conserved across *Arachis *genus, they may be valuable for comparative genome mapping and functional analysis of candidate genes. In addition, these markers may be potentially useful for study of pods traits because majority of these EST sequences were derived from pods at three developmental stages.

## Methods

### Plant materials and DNA extraction

In the present study, twenty-two accessions of cultivated peanut(*A.hypogaea *L.) corresponding to two subspecies (*hypogaea *and *fastigiata) *and sixteen accessions of wild species from seven sections of the genus *Arachis *were used (Additional File 2). The leaf samples of each accession were collected from Peanut Germplasm Bank located in Crops Research Institute, Guangdong Academy of Agriculture, Guangzhou, China. the genomic DNA was extracted as described by Sharma [56].

### Data mining for SSR marker

A total of 24,238 EST sequences including 20,160 developed by Guo et al (2008)[57] and 4078 retrieved from National Center of Biotechnology Information (NCBI) were used in this study. These ESTs were assembled using the TGICL program [36]. A Perl script known as MIcroSAtellite (MISA .) was used to mine microsatellites. In this work, SSRs were considered for primer design that fitted the following criteria: a minimum length of 14 bp, excluding polyA and polyT repeat, at least 7 repeat units in case of di-nucleotide and at least 5 repeat units for tri-, tetra-, penta- and hexa-nucleotide SSRs. Therefore, the paired numbers representing SSR motif length and the minimum repeat number in the MISA configuration file (misa.ini) were modified to 2–7, 3–5, 4–5, 5-5 and 6-5 (mono-type excluded).

### Primer design and PCR amplification

Using Primer Premier 5 program (Whitehead Institute for Biomedical Research, Cambridge, Mass), primers were designed based on the following core criteria: (1) melting temperature (Tm) between 52°C and 63°C with 60°C as optimum; (2) product size ranging from 100 bp to 350 bp; (3) primer length ranging from 18 bp to 24 bp with amplification rate larger than 80%; (4) GC% content between 40% and 60%. The parameters were modified when unsuitable primer pairs were retrieved by the program. PCR analysis was performed in a total volume of 20 μl with the following cycling profile: 1 cycle of 5 min at 94°C, an annealing temperature of 55°C for 35 cycles (1 min at 94°C, 30 s at 55°C, 45 s at 72°C) and an additional cycle of 10 min at 72°C. Each of the primer pairs was screened twice to confirm the repeatability of the observed bands in each genotype. PCR products were separated on 6% polyacrylamide denaturing gels. The gels were silver stained for SSR bands detection.

### Sequencing of PCR bands

The SSR alleles amplified in two cultivars and three wild species for EM-31 primer were individually cloned and sequenced. PCR amplification products were separated by 6% polyacrylamide gel and target allele bands were excised and dipped in 10 μl of nuclease free water for 30 min. Another round of PCR was made following the same protocol with recycled DNA as template. The second-round PCR products were separated in a 2% agarose gel and the target band was purified using TIANGEN Gel Extracting Kit (TIANGEN Inc. Beijing China). The purified PCR fragment from agarose gel was cloned using the Takara TA cloning kit pMD-18 (Takara, Dalian, China). The ligation product was transformed into competent *Escherichia coli *cells. The positive clones identified by PCR were sequenced by Invitrogen Company. The final edited sequences belonging to different genotypes were compared with the original SSR containing EST sequence using Omiga program [58], and the exported multiple sequence alignment was modified by Genedoc .

### Data scoring and statistical analysis

The allelic and genotypic frequencies were calculated for the samples analyzed. The genetic diversity of the samples as a whole was estimated based on the number of alleles per locus (total number of alleles/number of loci), the percentage of polymorphic loci (number of polymorphic loci/total number of loci analyzed) and polymorphism information content (PIC). The polymorphism was determined according to the presence or absence of the SSR locus. The value of PIC was calculated using the formula



where *P*_*i *_is the frequency of an individual genotype generated by a given EST-SSR primer pair and summation extends over *n *alleles.

## Authors' contributions

All authors read and approved the final manuscript. XL participated in conceiving the study and drafting the manuscript. XC participated in conceiving the study, sequence analysis and drafting the manuscript. YH participated in conceiving the study, the development of SSR markers and data analysis. HL developed the SSRs and designed the SSR primers. GZ and SL planted and collected the peanut materials. BG participated in the development of SSR markers.

## Supplementary Material

Additional File 1 **List of EST-SSR primers developed from cultivated peanut ESTs**. The file contains a table that lists primer names, repeat motifs, primer sequences, allele number and product length for the newly developed EST-SSR markers. Click here for file

Additional File 2 **List of cultivated peanut and wild species materials used in this study**. The file includes a table that lists the name, type, ploidy and origin of 22 genotypes of cultivated peanuts and 16 accessions of wild species tested in this study. Click here for file
